# Dietary Strategies in Postmenopausal Women with Chronic and Metabolic Diseases

**DOI:** 10.3390/nu16091329

**Published:** 2024-04-28

**Authors:** Tiffany M. Cortes, Monica C. Serra

**Affiliations:** 1 Sam & Ann Barshop Institute for Longevity & Aging Studies, Department of Medicine, University of Texas Health Science Center San Antonio, San Antonio, TX 78229, USA; cortest@uthscsa.edu; 2 South Texas Veterans Health Care System, Geriatric Research Education and Clinical Center, San Antonio, TX 78229, USA

As women age, their nutritional needs change, governed by changes in hormones, level of physical activity, and dietary intake. During and after menopause, there are reductions in the resting metabolic rate and changes in the body composition, including body weight and fat mass gain (particularly in the abdominal region) and loss of bone mineral density, connective tissue, and muscle mass [1]. These changes in metabolic function and body composition place postmenopausal women at elevated risk for chronic conditions, including cardiovascular diseases, type 2 diabetes mellitus, osteoporosis, dementia, depression, and cancer, compared to their younger/premenopausal counterparts [2].

This Special Issue of *Nutrients*, “Dietary Strategies in Postmenopausal Women with Chronic and Metabolic Diseases”, aimed to present articles focused on: (1) the metabolic pathways affected by the menopausal transition, (2) how dietary patterns in postmenopausal women influence chronic conditions (and vice versa), and (3) what is known about how dietary modification may influence the health of older women, particularly those with chronic conditions. This Special Issue collected five original and three review articles. These articles allowed the reader to (1) delve into the wide range of conditions that affect the menopausal transition and postmenopausal women, (2) understand how life changes impact these conditions and transitions, and (3) identify ways to mitigate the observed adverse changes.

A wide range of non-traditional therapies, including yoga, aromatherapy, and dietary supplements, have been evaluated to aid in adverse changes seen with menopause [3,4]. Three authors investigated the use of various dietary supplements as interventions to address adverse metabolic changes associated with estrogen deficiency. Pereira et al. [5] studied the effects of flaxseed and mulberry extracts on several metabolic outcomes in ovariectomized rats. Data presented showed that the extracts were rich in phenolic compounds and had high antioxidant activity. When consumed individually or combined, the extracts appeared to have similar effects as estrogen treatment (with varying magnitude) with regard to body weight, reproductive health, and lipid profiles. Further, the authors observed minimal hepatic or renal adverse effects. These findings suggested the need for further clinical evaluation, as supplementation with flaxseed and/or mulberry extracts could offer health benefits and serve as a nutraceutical alternative to alleviate the negative effects resulting from decreased or absent estrogenic action in the body. Next, DiStefano [6] reviewed the role of choline, soy isoflavones, and probiotics as adjuvant treatments for nonalcoholic fatty liver disease (NAFLD) in postmenopausal women. The author reported the following findings: (1) choline deficiency, particularly in postmenopausal women, can lead to liver dysfunction, and estrogen deficiency may exacerbate this risk, suggesting potential benefits from menopausal hormone therapy and increased choline intake; (2) soy isoflavones, found in soybeans, are associated with liver health benefits, and may offer unique advantages for postmenopausal women due to their estrogen-like effects on the metabolism; and (3) probiotics were linked to improved liver health in individuals with NAFLD and gut dysbiosis, though studies specifically focused on postmenopausal women were lacking. Overall, the author suggested that targeted nutritional interventions, including choline, soy isoflavones, and probiotics, may hold promise for preventing and treating NAFLD in postmenopausal women, but further research is needed to confirm their efficacy. In Bauset et al.’s review [7], the impact of nut consumption on the risk of metabolic syndrome associated with menopause was evaluated. The authors reported that although nuts are a highly caloric food, their high proportions of unsaturated fat, monounsaturated fatty acids, and polyunsaturated fatty acid, as well as components of phenolic compounds, phyoesterol, antioxidants, and fiber, may contribute to beneficial metabolic changes through reductions in oxidative stress and inflammation and changes in the microbiota. The authors proposed that nut ingestion may have beneficial effects on altered lipid profiles, carbohydrate metabolism, and fat accumulation associated with menopause, but the need for further research remains. Together, the data from these three studies suggest promising avenues for the use of dietary supplements and nutritional interventions to mitigate adverse metabolic changes associated with estrogen deficiency and improve the overall health outcomes of postmenopausal women.

Dietary protein intake for postmenopausal women has been recommended to be 1.1–1.5 g/kg/day, which is higher than the general adult recommended dietary allowance of 0.8 g/kg/day, and these increases may have beneficial effects on muscle and bone health [8,9]. Two studies evaluated the impact of protein supplementation on body composition in postmenopausal women. Kuo et al. [10] presented their review and meta-analysis on the effects to whey protein (WP) supplementation by itself and in combination with resistance training (RT) for postmenopausal women, based on the knowledge that RT and WP consumption are established interventions providing a positive effect on muscle health in general populations. The authors summarized that WP supplementation with RT significantly enhanced lower limb lean mass gain and bicep curl strength, without RT significantly reduced protein intake, and with or without RT promoted fat mass loss. However, they acknowledge the need for further research with larger sample sizes and uniform outcome measures. Norton et al. [11] investigated if a milk based protein matrix (MBPM) ingested in the evening might take advantage of the nocturnal peak of bone remodeling and have a positive effect on bone health in 83 postmenopausal women with osteopenia. MBPM, compared to isoenergetic maltodextrin control, ingested at bedtime over 24 weeks had a decline of bone resorption marker C terminal cross linked telopeptide of type 1 collaged, but not on clinical measures, including bone mineral density or trabecular bone score. These authors concluded that a chronotherapeutic approach to MBPM supplementation may show promise on homeostatic bone remodeling in postmenopausal women at risk for degenerative bone disease. Overall, these two studies suggested the potential benefits of protein supplementation, particularly whey protein and milk-based protein matrices, in enhancing muscle health and bone remodeling among postmenopausal women, but further research with larger sample sizes and standardized outcome measures is needed to validate these promising results.

The prevalence of metabolic syndrome is 2–3 times higher in postmenopausal women compared to premenopausal women [12]. Additionally, lipid parameters may have an adverse change noted within one year after final menstrual period [13]. This data supports the importance of identifying factors that that may contribute to the increased cardiometabolic risk noted with postmenopausal women. Three studies in this issue evaluated how eating patterns in postmenopausal women affected their risk for metabolic disease. Kokkinopoulou et al. [14] evaluated whether differences in anthropometry, dietary intake, and metabolic syndrome (MetS) existed in postmenopausal women who followed Christian Orthodox Church (COC) religious fasting compared to those that were non-fasters. Characterized by abstinence from meat, dairy, and eggs, the COC fasting diet represents a plant-based to vegetarian diet. This study included 134 postmenopausal women, with 68 adhering to COC fasting for an average of 34 years. The authors reported that fasters showed significantly higher mean fat-free mass and hip circumference, and lower diastolic blood pressure (DBP), when compared to non-fasters. Fasters consumed significantly more monosaccharides, but had lower dietary intake of total, saturated, monounsaturated, and polyunsaturated fats, trans and omega-6 fatty acids, and cholesterol, when compared to non-fasters. Biochemically, fasters had lower blood glucose concentrations and MetS prevalence. The authors concluded that postmenopausal women adhering to COC fasting exhibited differences in anthropometry, dietary intake, and metabolic parameters, which suggested potential cardiovascular benefits associated with this dietary pattern. Hooper et al. [15] studied the psychosomatic, cardiometabolic, body composition, and physical function characteristics of 21 postmenopausal women with current binge eating disorder (BED). They report limited available data to date on the prevalence of binge eating among older women, and its potential impact on physical and mental health. Pertinent findings included high rates of comorbidities, including depression, anxiety, sleep problems, and severe menopausal symptoms. Cardiometabolic health indicators such as glucose and cholesterol concentrations also were noted to be elevated. The authors suggested a need for increased awareness among healthcare professionals regarding the screening and diagnosis of BED in older populations, considering its association with other chronic conditions common in postmenopausal women. Further, they suggested the need for more research into effective treatments for BED in older women, considering their exclusion from existing evidence-based treatments. Finally, Noll et al. [16] evaluated whether there are differences in menopausal symptoms and food consumption in 274 postmenopausal women before and during the COVID-19 pandemic. They reported that, during the pandemic, there was increased consumption of sweet foods and sugar-sweetened beverages and decreased intake of milk, dairy products, and processed foods among postmenopausal women. Despite a decrease in energy and macronutrient intake, the quality of the diet did not improve. However, postmenopausal women reported a lower intensity of menopausal symptoms, including vasomotor symptoms, during the pandemic compared to before it. As suggested by the authors, these findings highlight the need for interventions to optimize dietary intake and mitigate the impact of the pandemic on postmenopausal women’s health. Combined, these three studies examining the impact of dietary choices and menopausal symptoms underscored the necessity for targeted interventions to promote healthier lifestyles in this vulnerable population.

In summary, this collection of research highlights the crucial need for tailored interventions to address the evolving nutritional needs and health challenges faced by postmenopausal women. It is imperative for healthcare systems to adapt and prioritize the long-term health of postmenopausal women through comprehensive and individualized care approaches. Strategies that support healthier dietary habits and mental well-being are essential for enhancing the overall quality of life and health of women as they age. By advancing our understanding and implementing comprehensive care approaches, we can empower postmenopausal women to navigate these transitions with greater vitality and longevity.
